# Root traits in response to frequent fires: Implications for belowground carbon dynamics in fire-prone savannas

**DOI:** 10.3389/fpls.2023.1106531

**Published:** 2023-03-07

**Authors:** Yong Zhou

**Affiliations:** ^1^ Department of Wildland Resources, Utah State University, Logan, UT, United States; ^2^ Ecology Center, Utah State University, Logan, UT, United States

**Keywords:** savannas, frequent fire, root functional trait, trait variation, belowground carbon allocation, soil carbon storage

## Abstract

Predicting how belowground carbon storage reflects changes in aboveground vegetation biomass is an unresolved challenge in most ecosystems. This is especially true for fire-prone savannas, where frequent fires shape the fraction of carbon allocated to root traits for post-fire vegetation recovery. Here I review evidence on how root traits may respond to frequent fires and propose to leverage root traits to infer belowground carbon dynamics in fire-prone savannas. Evidently, we still lack an understanding of trade-offs in root acquisitive vs. conservative traits in response to frequent fires, nor have we determined which root traits are functionally important to mediate belowground carbon dynamics in a frequently burned environment. Focusing research efforts along these topics should improve our understanding of savanna carbon cycling under future changes in fire regimes.

## 1 Introduction

**Figure 1:**
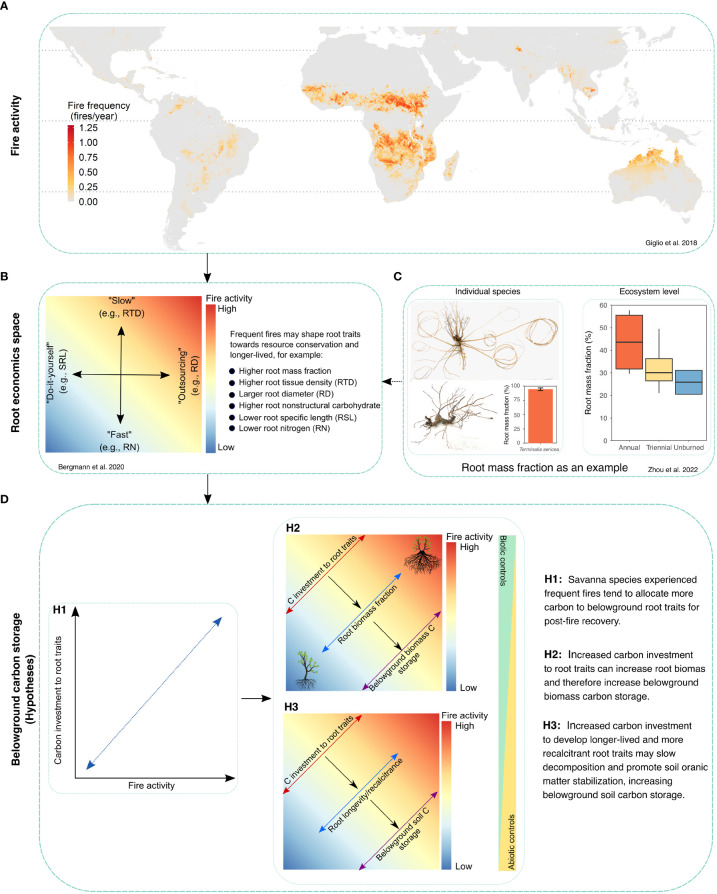
Linking root functional trait to understand belowground carbon dynamics in fire-prone savannas. Panel **(A)** shows fire frequency (fires/year) across tropical and subtropical savannas. Dataset were from the Moderate Resolution Imaging Spectroradiometer (MODIS) burned area product (Giglio et al., 2018). Panel **(B)** shows potential response of root traits to frequent fires in savannas based on root economics space (Bergmann et al., 2020). Frequent fires may shape root traits toward resource conservation and longer-lived, for example, higher root tissue density, higher root mass fraction, higher nonstructural carbohydrate, and lower root nitrogen content. Panel **(C)** shows an example of root mass fraction response to frequent fires at the individual species and ecosystem level (Zhou et al., 2022). Root mass fraction of *Terminalia sericea* resprouters that experienced annual fires can reach up to 95%, and savannas experienced more frequent fires have higher root mass fraction at the ecosystem level than less and unburned ones in Kruger National Park, South Africa. Panel **(D)** shows a conceptual framework linking root traits to understanding belowground carbon storage in fire-prone savannas. Savanna species experiencing more frequent fires are expected to allocate more carbon for root traits (H1), which is hypothesized to lead to a higher belowground biomass carbon storage (H2) as well as soil carbon storage (H3).

Savannas occupy *ca.* 20% of the Earth’s land surface and account for *ca.* 30% of the terrestrial net primary production, contributing significantly to the terrestrial carbon cycle (Scholes and Archer, 1997; Grace et al., 2006). Savannas are fire-prone ecosystems and tropical savannas account for *ca.* 70% of the global burned area annually (Giglio et al., 2018) (**Figure 1A**). Frequent fires, as a unique characteristic of savannas, not only shape aboveground vegetation physiognomic composition and biomass (Staver et al., 2011), but also influence the fraction of carbon allocated to belowground for post-fire vegetation recovery (Teixeira et al., 2022; Zhou et al., 2022), making it difficult to predict how belowground carbon storage will change alongside aboveground carbon. This unpredictable contribution of belowground carbon to whole-ecosystem carbon storage represents a major unresolved challenge for quantifying the contributions of savannas in the terrestrial carbon cycle.

Belowground carbon allocation is primarily driven by roots, and therefore root functional traits play an essential role in determining spatial and temporal dynamics of belowground carbon inputs and cycling (Freschet et al., 2021). Recent advances in trait-based approaches have shown promising potential to understand ecosystem carbon cycling (de Deyn et al., 2008; Bardgett, 2017; Laliberté, 2017; Poirier et al., 2018; Freschet et al., 2021; Weigelt et al., 2021; Hallett et al., 2022). In view of this, here I first summarize how root traits may respond to frequent fires (**Figure 1**, **Table 1**) and then propose the integration of root traits to improve our understanding of belowground carbon dynamics in fire-prone savannas (**Figure 1**), while identifying challenges and potential resolutions.

**Table 1:** Summary of resource acquisitive (A) and conservative (C) root traits in response to frequent fires and their implications for belowground carbon dynamics in fire-prone savannas.

Root traits	Potential functions for post-fire recovery	Potential trend in response to fire *	Implications to belowground carbon dynamics**	Fire studies in savannas
**Root nitrogen (A)**	• Roots with higher nitrogen content have higher metabolic rates but a shorter lifespan. • Have a fast resource return on investment	Decrease [*more studies needed*]	Decreasing root nitrogen may decrease root decay and increase soil carbon storage	Pellegrini et al., 2021 (a global synthesis but includes savannas)
**Specific root length (A)**	• Roots with higher specific root length have higher rates of resource uptake but a shorter lifespan	Increase [*more studies needed*]	Increasing SRL may increase root decay and decreas soil carbon storage	le Stradic et al., 2021; Teixeira et al., 2022
**Root mycorrhizal colonization (A)**	• Facilitates nutrient acquisition	Decrease [*more studies needed*]	Decreasing root mycorrhizal colonization may enhance root decay and carbon loss	Hartnett et al., 2004
**Root nonstructural carbohydrate (C)**	• Supports post-fire vegetation regeneration and reproduction	Increase	Increasing root NSC can increase carbon storage in root biomass	Wigley et al., 2009, Wigley et al., 2019; Clarke et al., 2016
**Root sucker (C)**	• Facilitates post-fire vegetation regeneration	Increase	Increasing root sucker may increase carbon storage in root biomass	Charles-Dominique et al., 2015; Charles-Dominique et al., 2017; Hoffman, 1998; Salazar and Goldstein, 2014
**Root bud bank (C)**	• Facilitates post-fire vegetation regeneration	Increase	Increasing root bud bank may increase carbon storage in root biomass	see Pausas et al., 2018 for a review
**Root mass fraction (A/C)**	• Higher root mass fraction supports more nonstructural carbohydrate storage, root suckers and bud banks • Higher root mass fraction facilitates resource acquisition	Increase	Increasing root mass fraction increases carbon storage in root biomass	Wigley et al., 2019; le Stradic et al., 2021; Teixeira et al., 2022; Zhou et al., 2022
**Root tissue density (A/C)**	• Roots with denser tissue are more resistant to decay, protecting longevity of belowground storage organs. • Conserve resources and have a slow resource return on investment	Inconclusive [*more studies needed*]	***Inconclusive***, but increasing root tissue density may suppress root decay and carbon loss	le Stradic et al., 2021; Teixeira et al., 2022
**Root diameter (A/C)**	• Roots with thicker diameter may facilitate mycorrhizal colonization. • Negatively associated with specific root length	No effect [*more studies needed*]	***No effect***, but increasing root diameter may suppress root decay and carbon loss	le Stradic et al., 2021

* The potential trend of root trait in response to frequent fires is based on studies listed within the right column.

** Implications to belowground carbon dynamics is based on the potential trend of root trait in response to frequent fires.

## 2 Root traits response to fires in savannas: Knowns and unknowns

Fire is frequent in tropical savannas (**Figure 1A**). Although frequent fires may directly affect certain root traits (*e.g.*, lifespan) through fire-induced changes in soil environment variables (*e.g*., temperature, moisture) (Michaletz and Johnson, 2007), fire impacts on root traits are most likely associated with the post-fire vegetation recovery. To survive frequent fires, a majority of savanna plant species, especially woody species, are able to resprout from belowground reserves despite considerable damage to their aboveground compartments (*i.e.*, top-kill) (Bond and Midgley, 2003). This ability to repeatedly resprout is dependent upon a set of adaptive traits and their carbon reserves (Keeley et al., 2011; Clarke et al., 2013), especially belowground root traits associated with resource conservation and acquisition (Paula and Pausas, 2011; Boonman et al., 2020). However, our current knowledge regarding root traits in response to frequent fires is mostly centered around their trait functions related to carbon conservation rather than nutrient acquisition (**Table 1**).

On the root economics space (Bergmann et al., 2020), savanna plant species may tend to allocate more carbon belowground and to develop root traits that are long-lived and well-protected in a frequently burned environment (**Figure 1B**). There is robust evidence from both experimental studies and field observations that savanna plant species experiencing frequent fires have a larger root mass fraction than those growing free from fires both at the individual species level and ecosystem level (**Figure 1C**, **Table 1**) (Boonman et al., 2020; Teixeira et al., 2022; Zhou et al., 2022). Likewise, savanna plant species persisting through frequent fires generally store large amounts of non-structural carbohydrates in specialized root organs (*e.g.*, lignotubers) (Wigley et al., 2009; Clarke et al., 2016; Diaz-Toribio and Putz, 2021), which are critical to support root bud banks and the ability of root sucker for post-fire resprouting (Charles-Dominique et al., 2015; da Silva et al., 2020). Similar conservative strategies have also been reported for woody plant resprouters in fire-prone Mediterranean ecosystems (Paula and Pausas, 2011). Additionally, although limited in evidence, savanna plant species withstanding frequent fires for longer periods of time are found to have roots with denser tissue and lower nitrogen content (Pellegrini et al., 2021; Teixeira et al., 2022), indicating that savanna species are more likely construct long-lived roots in a frequently burned environment.

This increased belowground biomass investment may also lead to a shift in root traits associated with resource acquisition (le Stradic et al., 2021), because frequent fires volatilize plant essential nutrients (especially nitrogen) in savannas that are already considered as nutrient-limited. To fulfil this high demand for belowground nutrients during the post-fire recovery, savanna plant species experiencing frequent fires may either increase root exploration and/or exploitation of nutrients within the soil space (for example, high specific root length) or increase carbon investment into mycorrhizal colonization to acquire nutrients collaboratively. While some studies have found evidence for this idea (le Stradic et al., 2021; Teixeira et al., 2022), others have suggested that there are trade-offs between acquisition and conservation on the root economics space (**Figure 1B**) and that the optimization towards one or the other depends on a variety of factors, such as nutrient availability (Tomlinson et al., 2012; Boonman et al., 2020). To resolve this inconsistency, further systematic studies on intraspecific variation in resource conservation and acquisition traits across a fire frequency gradient for longer periods of time are needed, under the same controlled environmental conditions to exclude the influence of other factors. Additionally, our current understanding of root traits in response to fires are centered on fire frequency, it remains largely unknown whether and how other aspects of fire characteristics (*e.g.*, fire intensity and severity) would change these trade-offs.

## 3 Linking root traits to belowground carbon dynamics in fire-prone savannas: Challenges and resolutions

This increased carbon investment into root traits for post-fire recovery could have significant impacts on belowground carbon storage and dynamics in fire-prone savannas (**Figure 1D**). However, it remains unknown how and to what extent this increased carbon investment contributes to the current large uncertainty in the magnitude and direction of fire impacts on belowground carbon storage (especially soil carbon storage) in savannas (Pellegrini et al., 2018; Teixeira et al., 2022; Zhou et al., 2022). A potential way to close this gap is to integrate root trait-based approaches to understand savanna belowground carbon cycling, which has been applied to other ecosystems and is a rapidly advancing frontier both conceptually and empirically (e.g., de Deyn et al., 2008; Bardgett, 2017; Poirier et al., 2018; Han and Zhu, 2021; Jiang et al., 2021).

Several mechanisms may be at play in terms of linking root traits to belowground carbon cycling in fire-prone savannas. On the one hand, increased carbon investment into root traits for post-fire recovery can substantially increase belowground root productivity with a net outcome of increased root mass fraction and belowground biomass carbon storage (**Figure 1D**). However, one important research challenge is to improve the accuracy of root biomass estimates across a large range of savanna ecosystems experiencing frequent fires. For example, in a few studies that consider root biomass, root biomass is arbitrarily assumed to increase in proportion to aboveground biomass, yielding constant root-to-shoot ratios across fire frequencies (Tilman et al., 2000; Pellegrini et al., 2015). However, this assumption is obviously problematic as savanna plant species experiencing more frequent fires allocate more biomass belowground (**Figure 1C**) (Wigley et al., 2009; Zhou et al., 2022). One promising but more challenging solution is to adopt the compartment-based approach, which categorizes belowground plant organs into acquisitive (e.g., fine roots, mycorrhizal associations) and non-acquisitive (*e.g.*, clonal and storage organs) compartments (Klimešová et al., 2018). This approach has shown practical potential to advance the understanding of belowground biomass allocation and turnover in disturbed ecosystems. However, despite the reduced dimension to two compartments may shed light on the development of compartment-specific biomass allocation algorithms based on species identity, fire frequency, and other environmental factors, it does not necessarily mean that there will be a reduction in the number of root functional traits measured.

On the other hand, increased carbon investment to develop long-lived and well-protected root traits in a frequently burned environment may increase root recalcitrance to decomposition and contribute to soil organic matter stabilization (**Figure 1D**). Savanna plant species withstanding frequent fires may tend to construct dense, thick, low nitrogen content roots (Pellegrini et al., 2018; Teixeira et al., 2022). There is a compilation of published studies suggesting that root decomposition rate is positively corrected with root nitrogen content and negatively with root tissue and root diameter (de Deyn et al., 2008; Zhou et al., 2014; Jiang et al., 2021). This retarded root decomposition may enhance the formation of particulate organic matter and promote soil organic matter stabilization and persistence. However, our understanding of linking root traits to belowground carbon cycling is still limited to the application of a few easily measurable root traits (*e.g.*, root tissue density and specific root length) (le Stradic et al., 2021; Teixeira et al., 2022). These most commonly measured root traits may not be functionally important to the formation and stabilization of soil organic matter. For example, root exudates are not well-studied in fire-prone savannas (**Table 1**), but they can contribute up to a third of carbon inputs into soils and have been shown to accelerate the decay of organic matter (Keiluweit et al., 2015) and/or contribute to the formation of stable soil organic carbon (Sokol et al., 2019; Dijkstra et al., 2021). Additionally, root recalcitrance to decomposition is not the whole story of the formation and persistence of soil organic matter. Other root traits, such as mycorrhizal hyphae and the density of root hairs, also play significant roles in belowground soil carbon dynamics by facilitating the formation of soil aggregates that enhance the protection of occluded carbon from microbial attack (Rillig et al., 2015; Hallett et al., 2022). Therefore, the priority is to determine how underrepresented traits influence belowground soil carbon cycling and how best to measure and represent their contributions to different aspects of soil organic matter formation and stability in a frequently burned environment. Furthermore, because the stability and persistence of organic carbon is closely associated with physicochemical characteristics inherent to soil minerals and other abiotic factors (Possinger et al., 2020), further studies should also consider site differences in these abiotic factors that can substantially influence the preservation of root-derived carbon in soils (**Figure 1D**).

Evidently, responses of root traits to frequent fires have significant implications to savanna belowground carbon cycling and therefore the integration of root traits may provide additional power in reducing the uncertainty of predicting the contribution of belowground carbon to whole-ecosystem carbon storage in fire-prone savannas. However, many studies thus far have modeled responses of savanna belowground carbon storage (mostly soil organic carbon storage) as a function of fire frequency (Coetsee et al., 2010; Pellegrini et al., 2015), or at most add another layer of abiotic factors, such as soil texture (Zhou et al., 2022). In view of trait-based approaches (Díaz et al., 2007), these models can be further extended to include community weighted trait mean, trait functional diversity, and their interaction terms with fire frequency. For example, a recent study, which examined how fire regimes affect ecosystem carbon exchange through functional diversity modifications in a tropical savanna, found that fire can promote root trait functional diversity and enhance soil organic carbon storage (Teixeira et al., 2022). This study demonstrates the feasibility and benefits of trait-based approaches in fire-prone savannas and relevant changes in ecosystem carbon cycle response to frequent fires are manifested through changes in root trait functional diversity. Despite this, however, critical challenges are still posed by limited data availability on how root traits response to frequent fires. Although many joint worldwide efforts have made in recent years to create root trait database (*e.g*., the Global Root Traits database) (Guerrero-Ramírez et al., 2021), information on root trait plasticity and/or life history of the plant are still lacking. However, acquiring such information generally needs to take into account the variation in root traits over time, which requires long-term commitment of funding resources and personnel. Alternatively, future work may take advantage of long-term prescribed burning experiments with known fire history across savannas (Pellegrini et al., 2015; Pellegrini et al., 2021) to derive predictable relationships among fire frequency, root trait variation, ecosystem carbon storage and productivity. Ultimately, this may facilitate the prediction and modelling of savanna carbon dynamics under future changes in fire regimes.

## 4 Conclusion

Using fire-prone savanna ecosystems as an example, I have highlighted the substantial influence of frequent fires on intraspecific variation in root traits with significant implications for savanna carbon dynamics (**Figure 1**, **Table 1**). While trait-based approaches may provide promise for integrating root traits to understand savanna carbon cycling, the key challenges lie with the following 1) the trade-offs in the carbon allocation to develop acquisitive or conservative root traits in response to frequent fires; 2) the identification of functionally important root traits rather than easily measurable ones in terms of determining belowground carbon storage and dynamics in fire-prone savannas; and 3) the lack of data on root trait variation of savanna plant species with known fire history. Addressing these challenges could not only help us to better understand savanna belowground carbon storage and dynamics in a world with predicted changes in fire regimes, but also improve the application of root trait-based approaches to predict ecosystem functioning in other ecosystems that experience regime changes (*i.e.*, drought, pathogen, and nitrogen deposition).

## Author contributions

The author confirms being the sole contributor of this work and has approved it for publication.
